# Genomic analysis and antimicrobial resistance in human- and poultry-derived *Campylobacter jejuni* isolates from Hangzhou, China

**DOI:** 10.3389/fmicb.2025.1599555

**Published:** 2025-06-23

**Authors:** Mingfang Yang, Xin Wang, Luping Zheng, Yongli Zhu

**Affiliations:** ^1^Department of Microbiology, Tonglu County Center for Disease Control and Prevention (Tonglu County Health Inspection Center), Hangzhou, China; ^2^Medical Laboratory, Hangzhou First People’s Hospital Tonglu Campus, Hangzhou, China

**Keywords:** *Campylobacter jejuni*, antimicrobial resistance, virulence genes, multilocus sequence typing, whole genome sequencing

## Abstract

*Campylobacter jejuni* (*C. jejuni*) is a zoonotic pathogen and is the most prevalent foodborne pathogen globally. The increasing antimicrobial resistance and gene mutation pose a threat to public health and trigger us to enhance surveillance. This study illustrated the antimicrobial resistance profiles, virulence factors, and multilocus sequence typing (MLST) profiles of 47 *C. jejuni* isolates collected from human stool and raw poultry meat samples between 2022 and 2023 in Hangzhou, China. Notably, 88.9% (16/18) human-derived and 82.9% (24/29) poultry-derived *C. jejuni* displayed multidrug resistance (MDR) profiles, nalidixic acid + ciprofloxacin + tetracycline was prevalent among them. Relatively high rates of resistance to florfenicol were observed in *C. jejuni*, 38.9% (7/18) from human sources and 44.8% (13/29) from poultry sources. Comprehensive Antibiotic Resistance Database (CARD) and ResFinder database showed *gyrA* (*T86I*) was the dominant factor in quinolones resistance while a rare *gyrA* (*T86V*) was found in one poultry-derived *C. jejuni*. All 37 tetracycline-resistant *C. jejuni* strains harbored the *tet(O)* gene. All 20 florfenicol-resistant *C. jejuni* did not have any related genes. Several key virulence factor genes associated with adherence (*cadF, pebA, jlpA*, and *porA*), invasion (*CiaB* and *CiaC*), capsule biosynthesis/transport genes (*kpsF, kpsD, kpsE, kpsM, kpsC*, and *Cj1419c*) and lipooligosaccharide (LOS) biosynthesis genes (*Cj1135, waaV, waaF, htrB, gmhA*, and *gmhB*) were conserved in *C. jejuni*. MLST analysis revealed high genetic diversity, identifying 28 sequence types (STs), including 3 novel STs, 20 of which belonged to 10 clonal complexes (CCs), and 8 were unassigned. CC-464 and CC-21 strains carried the most virulence genes, correlating with clinical severity, yet represent a minority in local isolates. The most abundant CCs were CC-443 (21.3%, 10/47) and CC-574 (19.1%, 9/47), mainly isolated from poultry. These findings highlight that *C. jejuni* isolates in Hangzhou had high genetic diversity and MDR, CC-443 and CC-574 were the predominant strains. It is necessary to monitor human-poultry transmission and emerging resistance phenotypes.

## Introduction

*Campylobacter*, a Gram-negative foodborne pathogen, is a major cause of diarrheal disease in developed and developing countries (Kaakoush et al., 2015). World Health Organization (WHO) reported in 2020 that *Campylobacter* was 1 of 4 key global causes of diarrheal diseases (World Health Organization, 2020). Among the species, *C. jejuni* has higher detection and clinical relevance, almost all Campylobacteriosis cases are attributed (Khairullah et al., 2024), which mainly asymptomatically colonizes the intestinal tracts of poultry and livestock. Thus, *C. jejuni* can be detected in contaminated environments, undercooked poultry, and livestock meats.

Outbreaks caused by *C. jejuni* are not uncommon worldwide. In general, abdominal pain and diarrhea are the common symptoms, most patients heal themselves within 1 week, but antimicrobial treatment is recommended in invasive cases, macrolides, and quinolones are the first choices (Same and Tamma, 2018). However, the popularity and excellent antibacterial ability of quinolones have led to their widespread use since they were first introduced in clinical treatment (Bush et al., 2020). Many reports have shown significant increases in the rates of both quinolones resistance (Veltcheva et al., 2022) and other antimicrobial resistance (AMR) (Phu et al., 2025). Furthermore, rare but severe neurological complications such as Guillain-Barré syndrome (GBS) underscore the pathogen’s public health significance, prompting active surveillance by agencies like the Center for Disease Control and Prevention (CDC).

With increasing gene mutation and AMR of *C. jejuni*, comprehensive research can be performed through whole genome sequencing (WGS) due to its high accuracy, good repeatability, and high resolution (Jia et al., 2025). In China, some *C. jejuni* foodborne outbreaks have been reported in Beijing (Li Y. et al., 2020), Lishui (Ge et al., 2024), Zhoushan (Chen et al., 2020), and other places. In our area, multiple food poisoning incidents (Yu et al., 2020) caused by *C. jejuni* have occurred. However, there remains a lack of comprehensive understanding regarding the epidemiological characteristics of *C. jejuni* in this area. Therefore, in this study, we conducted WGS on *C. jejuni* strains isolated from two sources: fecal specimens of diarrhea patients and poultry products commonly consumed by the local population. Through this approach, we aim to elucidate the genetic diversity, AMR profiles, and virulence factor distribution of *C. jejuni* within our area to provide support for control and prevention of *C. jejuni* foodborne outbreaks.

## Materials and methods

### Strain source

Eighteen strains of *C. jejuni* were isolated from 186 diarrhea patients in a sentinel hospital in Tonglu District of Hangzhou between 2022 and 2023. Twenty-nine strains of *C. jejuni* were isolated from 150 raw poultry meat samples collected at 2 farmers’ markets and 2 supermarkets in Tonglu District of Hangzhou. The isolates were systematically designated with “B” prefixes for human-derived strains, “J” prefixes for chicken-derived strains, and “Y” prefixes for duck-derived strains.

### Microbial collection and cultivation

Fresh diarrheal stool samples were collected using sterile swabs, immediately placed into Cary-Blair transport medium, and delivered to the laboratory within 24 h under 4°C (National Health and Family Planning Commission of the People’s Republic of China, 2007). Raw meat specimens were sealed in sterile homogenization bags and transported within 2 h. *Campylobacter* spp. isolation based on the filter-based method using a *Campylobacter* isolation kit (ZC-CAMPY-001 for human stool, ZC-CAMPY-002 for raw poultry meat, Tsingtao Sinova-HK Biotechnology Co., Ltd., Qingdao, China). For human stool samples, 3–5 g Cary-Blair agar with stool was aseptically transferred into 4 ml of growth-promoting enrichment broth and homogenized using a vortex mixer to ensure complete suspension. For raw poultry meat, the entire fresh carcass (approximately 1 kg) was placed into a sterile homogenization bag containing 500 ml of buffered peptone water (BPW), manually massaged for 5 min, and subsequently removed; 2 ml of the resulting BPW suspension was then added to 4 ml of enrichment broth. All broths were incubated at 42°C under microaerobic conditions (85% N_2_, 10% CO_2_, and 5% O_2_) for 24 h. Enriched broth was subsequently filtered through membrane filter (0.45 μm) onto Columbia Blood Agar and Karmali Agar. After 45–60 min of adsorption, membranes were aseptically removed. Plates were incubated at 42°C under microaerobic atmosphere (85% N_2_, 10% CO_2_, and 5% O_2_) for 48 h. Suspected single colonies (moist, spreading morphology) were subcultured on Columbia Blood Agar and followed by identification using Bacterial Identification System (VITEK 2 Compact, bioMérieux, France) and qPCR (*Campylobacter jejuni* and *Campylobacter coli* fluorescent PCR Test Kit (Lot number: T202303004), Jiangsu Bioperfectus Technologies Co., Ltd., Jiangsu, China). Standard strain ATCC 33560 was included in each batch.

### Antimicrobial susceptibility testing

Well-isolated colonies of *C. jejuni* strain from each positive sample (including both 18 human-derived and 29 poultry-derived isolates) were selected from agar plate and suspended in 3 ml of 0.85% NaCl solution to achieve a 0.5 MCF bacterial suspension. The ATCC 33560 reference strain was included in parallel as a quality control throughout the procedure. The minimum inhibitory concentration (MIC) of *C. jejuni* against erythromycin (ERY), azithromycin (AZI), nalidixic acid (NAL), ciprofloxacin (CIP), gentamicin (GEN), streptomycin (STR), chloramphenicol (CHL), florfenicol (FLO), tetracycline (TET), telithromycin (TEL), and clindamycin (CLI) was obtained using antibiotics MICs of *Campylobacter* detection kit (ZC-AST-001, Tsingtao Sinova-HK Biotechnology Co., Ltd., Qingdao, China). After microaerobic (85% N_2_, 10% CO_2_, and 5% O_2_) incubation at 42°C for 24 h, the growth of *C. jejuni* in each well was observed. MICs were interpreted according to the Clinical and Laboratory Standards Institute (CLSI) and the National Antimicrobial Resistance Monitoring System (NARMS) breakpoint for ERY (*R* ≥ 32 μg/ml), AZI (*R* ≥ 1 μg/ml), NAL (*R* ≥ 32 μg/ml), CIP (*R* ≥ 4 μg/ml), GEN (*R* ≥ 4 μg/ml), STR (*R* ≥ 16 μg/ml), CHL (*R* ≥ 32 μg/ml), FLO (*R* ≥ 8 μg/ml), TET (*R* ≥ 16 μg/ml), TEL (*R* ≥ 8 μg/ml), and CLI (*R* ≥ 1 μg/ml).

### Whole genome sequencing

DNA was extracted using MagPure Bacterial DNA Kit (D6361-02, Guangzhou Magen Biotechnology Co., Ltd., China). DNA concentration was determined via Qubit4.0 (Q33226, Thermo Fisher Scientific Inc.). DNA integrity was assessed by 1% agarose gel electrophoresis. Genomes were sequenced with the Illumina NovaSeq 6000 platform at Sangon Biotech [Sangon Biotech (Shanghai) Co., Ltd., China]. After sequencing, raw reads were filtered via Trimmomatic (v0.36) (Bolger et al., 2014) by removing adaptors and low-quality reads, then clean reads were obtained. Genome assembly was done using SPAdes (v3.5.0) (Bankevich et al., 2012) and the Gapfiller (v1.11) (Nadalin et al., 2012) was used to fill gaps. The Supplementary Table 1 summarized genome assembly metrics. Gene predictions and annotations were generated using the Prokka (Version 1.10) (Seemann, 2014) and the National Center for Biotechnology Information (NCBI) database.

### Multilocus sequence typing and phylogenetic analysis

Seven house-keeping genes aspatase (*aspA*), glutamine synthetase (*glnA*), citrate synthase (*gltA*), serine hydroxymethyltransferase (*glyA*), phosphoglucomutase (*pgm*), transketolase (*tkt*), and ATP synthase alpha subunit (*uncA*) DNA sequences from each *C. jejuni* isolate were submitted to the PubMLST database^1^ for analysis to obtain allelic profile. ST and CC were identified after submitting alleles. A total of 33 *C. jejuni* reference genomes were retrieved from NCBI database using their annotated GenBank Assembly Accessions (GCA numbers). The core genome alignment was performed using Roary (v3.13.0) (Page et al., 2015) with annotated GFF3 files. Homologous gene clusters were identified using a protein identity threshold of 90% and core genes defined as those present in ≥95% of strains. The resulting core gene concatenated alignment was used to construct a maximum-likelihood phylogenetic tree with FastTree (v2.1.11) (Price et al., 2010) under the GTR + CAT model. Branch support was assessed using Shimodaira-Hasegawa-like local support values. Visualization was implemented in ChiPlot.^2^

### Identification of antimicrobial resistance genes and virulence genes

Protein sequences were submitted to the CARD^3^ to predict the resistomes using Resistance Gene Identifier (RGI) tool with default parameters (perfect and strict hits, exclude nudge, and high quality/coverage). ResFinder^4^ was used to refine predictions under *C. jejuni*-specific settings, applying thresholds of ≥90% sequence identity and ≥80% minimum coverage. Protein sequences of *C. jejuni* isolates were compared against the Virulence Factor Database (VFDB)^5^ using BLASTP (Basic Local Alignment Search Tool for proteins). Significant hits were retained based on thresholds of ≤1 × 10^5^ for *E*-value, ≥40% for percent identity, and ≥50 amino acids for minimum alignment length. Virulence genes of all *C. jejuni* were analyzed. Data was visualized by ChiPlot (see text footnote 2).

## Results

### Antimicrobial resistance

The AMR phenotype of 47 *C. jejuni* isolates (18 patients and 29 raw poultry meats) was shown in Table 1. For 18 strains of *C. jejuni* from patients, none strain was resistant to macrolides (ERY and AZI), ketolactones (TEL), chloramphenicol (CHL), and aminoglycoside (GEN), but the resistance rates to quinolones (NAL and CIP) and tetracycline (TET) reached 94.4% (17/18) and 88.9% (16/18), respectively. Similarly, for 29 poultry-derived *C. jejuni* isolates, the high resistance rates to NAL, CIP, and TET reached 93.1% (27/29), 89.7% (26/29), and 72.4% (21/29) but showed sensitivity to ERY and CHL. It is noteworthy that the FLO resistance rate of *C. jejuni* from both sources accounted for nearly half and one strain was resistant to CLI.

**TABLE 1:** Antimicrobial resistance of 47 *Campylobacter jejuni* strains to 11 kinds of antibiotics.

Categorization	Antibiotic	Human-derived strains (*n* = 18)		Poultry-derived strains (*n* = 29)		Total (*n* = 47)	
		Number	Percentage (%)	Number	Percentage (%)	Number	Percentage (%)
Macrolides	ERY	0	0.0	0	0.0	0	0.0
	AZI	0	0.0	1	3.4	1	2.1
Quinolones	NAL	17	94.4	27	93.1	44	93.6
	CIP	17	94.4	26	89.7	43	91.5
aminoglycosides	GEN	0	0.0	1	3.4	1	2.1
	STR	1	5.6	6	20.7	7	14.9
chloramphenicol	CHL	0	0.0	0	0.0	0	0.0
	FLO	7	38.9	13	44.8	20	42.6
Tetracycline	TET	16	88.9	21	72.4	37	78.7
Ketolactones	TEL	0	0.0	2	6.9	2	4.3
Lincosamides	CLI	1	5.6	1	3.4	2	4.3

As illustrated in Table 2, 88.9% (16/18) of *C. jejuni* isolates from human sources and 82.8% (24/29) from poultry meats exhibited a MDR pattern, defined as concurrent resistance to three or more antibiotics (Bai et al., 2024). The prevalent MDR profile was NAL + CIP + TET (38.3%, 18/47), consistent with literature reports (Gao et al., 2023; Zhang L. et al., 2020), followed by NAL + CIP + FLO + TET (23.4%, 11/47). In contrast to the single human-derived *C. jejuni* strain resistant to five antibiotics, four poultry meat-origin strains exhibited resistance to the same number of antimicrobial agents. Poultry-origin isolates exhibited a broader spectrum of MDR, displaying 11 distinct resistance profiles, while human-origin isolates showed only 6 profiles.

**TABLE 2:** Multi-drug resistance (MDR) patterns detected among 47 *Campylobacter jejuni* strains.

MDR	Human-derived strains (*n* = 18)		Poultry-derived strains (*n* = 29)		Total (*n* = 47)	
	Number	Percentage (%)	Number	Percentage (%)	Number	Percentage (%)
NAL + CIP + STR + FLO + TET	1	5.6	3	10.3	4	8.5
NAL + CIP + FLO + TET + CLI	0	0.0	1	3.4	1	2.1
NAL + CIP + STR + TET	0	0.0	1	3.4	1	2.1
NAL + CIP + STR + FLO	0	0.0	1	3.4	1	2.1
AZI + GEN + STR + TEL	0	0.0	1	3.4	1	2.1
NAL + CIP + FLO + TET	6	33.3	5	17.2	11	23.4
NAL + CIP + TET	8	44.4	10	34.5	18	38.3
NAL + CIP + FLO	0	0.0	1	3.4	1	2.1
FLO + TET + TEL	0	0.0	1	3.4	1	2.1
NAL + CIP + CLI	1	5.6	0	0.0	1	2.1
NAL + FLO	0	0.0	1	3.4	1	2.1
NAL + CIP	1	5.6	4	13.8	5	10.6
TET	1	5.6	0	0.0	1	2.1

### Antimicrobial resistance gene analysis

Analysis of resistomes in CARD and ResFinder databases revealed an obvious correlation between phenotype and genotype. As shown in Table 3, all *C. jejuni* isolates carried *gyrA, cmeA*, and *cmeR* genes, as for quinolones, a majority of researches (Cobo-Díaz et al., 2021; Fernández-Palacios et al., 2024) indicate that *gyrA Thr86Ile* (*T86I*) is the most prevalent mutation. We found the T86I mutation in *gyrA* was predominant, detected in 91.5% (43/47). Strain J26 exhibited a rare *gyrA Thr86Val* (*T86V*) mutation while maintaining NAL resistance (MIC > 64) and CIP resistance (MIC = 32) (Supplementary Table 2). Three strains (B71, J116, and J117) had no mutation in the *gyrA* gene, while J117 showed resistance to NAL (MIC = 32) (Supplementary Table 2).

**TABLE 3:** Antimicrobial resistance gene carrying in 47 *Campylobacter jejuni* strains.

Categorization	Resistance genes	Human-derived strains (*n* = 18)		Poultry-derived strains (*n* = 29)		Total (*n* = 47)	
		Number	Percentage (%)	Number	Percentage (%)	Number	Percentage (%)
Quinolones	*gyrA*	18	100.0	29	100.0	47	100.0
	*gyrA T86I*	17	94.4	26	89.7	43	91.5
	*gyrA T86V*	0	0.0	1	2.1	1	2.1
Aminoglycosides	*APH(3′)-IIIa*	1	5.6	5	17.2	6	12.8
	*ant(6)-Ia*	1	5.6	6	20.7	7	14.9
	*aadE-Cc*	0	0.0	2	6.9	2	4.3
Tetracyclines	*tet(O)*	16	88.9	22	75.9	38	80.9
	*tet(O/M/O)*	5	27.8	3	10.3	8	17.0
	*tet(O/32/O)*	1	5.6	0	0.0	1	2.1
β-Lactams	*blaOXA-61*	9	50.0	13	44.9	22	46.8
	*blaOXA-184*	2	11.1	10	34.5	12	25.5
	*blaOXA-193*	9	50.0	13	44.8	22	46.8
	*blaOXA-450*	9	50.0	13	44.8	22	46.8
	*blaOXA-451*	9	50.0	13	44.8	22	46.8
	*blaOXA-452*	9	50.0	13	44.8	22	46.8
	*blaOXA-453*	9	50.0	13	44.8	22	46.8
	*blaOXA-460*	3	16.7	0	0.0	3	6.4
	*blaOXA-465*	0	0.0	2	6.9	2	4.3
	*blaOXA-489*	9	50.0	13	44.9	22	46.8
	*blaOXA-591*	2	11.1	2	6.9	4	8.5
	*blaOXA-594*	0	0.0	1	3.4	1	2.1
	*blaOXA-631*	0	0.0	2	6.9	2	4.3
	*blaTEM-1B*	0	0.0	1	3.4	1	2.1
Nucleoside antibiotic	*SAT-4*	1	5.6	4	13.8	5	10.6
Multidrug efflux pump and repressor	*cmeA*	18	100.0	29	100.0	47	100.0
	*cmeB*	9	50.0	14	48.3	23	48.9
	*cmeR*	18	100.0	29	100.0	47	100.0

**FIGURE 1:**
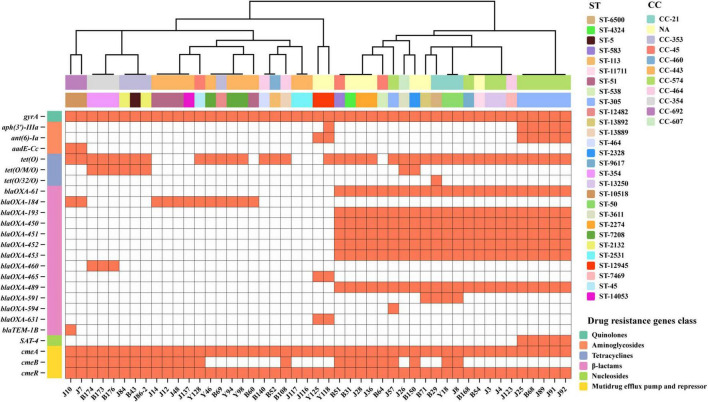
Antimicrobial resistance genes in 47 *C. jejuni* isolates. The heatmap displays the presence/absence of AMR genes in 47 *C. jejuni* isolates. Orange block indicates gene presence, white block indicates gene absence. AMR genes are categorized by antibiotic class (left), isolates are grouped by CC (top row) and ST (secondary row). The dendrogram is constructed based on a data matrix of gene presence/absence profiles.

All 37 tetracycline-resistant *C. jejuni* strains harbored the *tet(O)* gene encoding ribosomal protection proteins. Among them eight strains carried *tet(O)* in conjunction with mosaic variants [*tet(O/M/O)*], while B29 had a novel chimeric gene [*tet(O/32/O)*] (Figure 1). Nine isolates lacking known tetracycline resistance genes demonstrated susceptibility (Figure 1 and Supplementary Table 2). However, J89 harbored *tet(O)* gene was sensitive to tetracycline (MIC = 8) (Figure 1 and Supplementary Table 2).

β-Lactams resistant genes were identified by CARD and ResFinder databases, but the phenotype was not tested. As Table 3 showed, 14 β-lactams resistant genes were identified in this study, 7 β-lactamase genes (*blaOXA-61, blaOXA-193, blaOXA-450, blaOXA-451, blaOXA-452, blaOXA-453*, and *blaOXA-489*) were detected 46.8% (22/47) among the *C. jejuni* isolates, while bla*OXA-184* was 25.5% (12/47).

As for aminoglycosides, B68 carrying *APH(3′)-IIIa*, and *ant(6)-Ia* was resistant to STR (MIC > 64) while sensitive to GEN (MIC < 0.5) (Figure 1 and Supplementary Table 2). J7 and J10 carried *aadE-Cc*, J7 displayed low-level STR resistance (MIC = 16) while J10 was sensitive to STR (MIC = 8). J116 without any aminoglycoside resistance genes and macrolides resistance genes were resistant to GEN (MIC = 16), STR (MIC = 16), and AZI (MIC = 2) (Figure 1 and Supplementary Table 2). Similarly, no chloramphenicol resistance gene was found in all strains, but 20 strains were resistant to FLO.

### Virulence gene analysis

A total of 106 virulence-related genes were screened using VFDB database, categorized into 7 functional groups: adhesion/colonization (*n* = 4), invasive antigen proteins (*n* = 2), motility (*n* = 54), cytolethal distending toxin (CDT) regulation (*n* = 3), capsule biosynthesis and transport (*n* = 28), LOS synthesis (*n* = 14), and type IV secretion systems (T4SS, *n* = 1). All strains carried more than 77 virulence genes, with a maximum of 100 detected. Genes associated with adhesion/colonization (*cadF*, *pebA*, *jlpA*, and *porA*) and invasion (*ciaB* and *ciaC*) were detected in all *C. jejuni*, as well as 43 motility-related genes (*flgD, fliM, fliA, flhF, fliI, fliF, fliG, fliH, motB, motA, fliN, fliE, flgC, flgB, flaG, fliD, fliS, flaC, fliP, flaD, fliR, pseG, pseH, fliL, pflA, fliQ, cheW, cheV, cheY, pseC, pseF, pseI, fliK, eptC, rpoN, flgA, flgS, flgQ, flgP, fliW, maf4, flgJ*, and *flgM*) (Supplementary Table 3).

**FIGURE 2:**
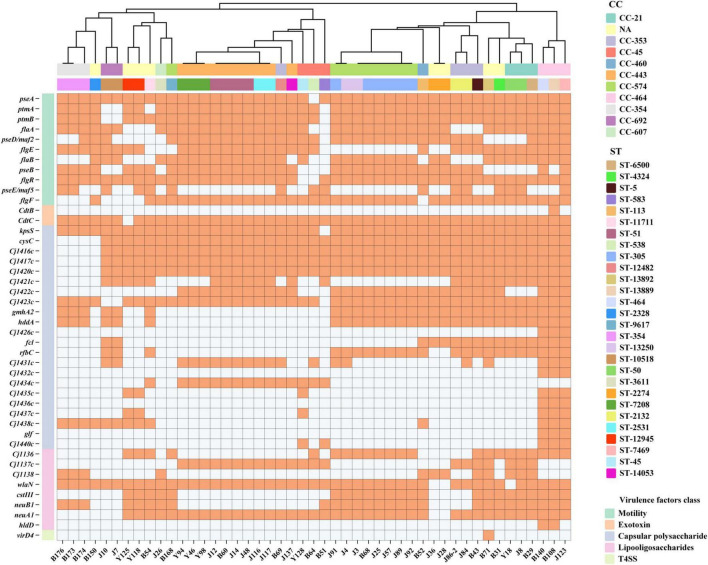
Remaining 44 virulence genes in 47 *C. jejuni* isolates. The heatmap displays the presence (orange) or absence (white) of virulence genes (left) in 47 *C. jejuni* strains (bottom). Isolates are grouped by CC (top row) and ST (secondary row). The dendrogram is constructed based on a data matrix of gene presence/absence profiles. Sixty-two virulence genes with 100% carrying rates were not annotated in the figure.

As shown in Figure 2, the presence of flagella encoded by *flaA* and *flaB* was detected in 83.0% (39/47) and 72.3% (34/47) of strains, respectively. Genes encoding CDT complex (*cdtA*, *cdtB*, and *cdtC*) showed differential distribution: *cdtA* was universally conserved, *cdtC* was present in 46 isolates, but only one single human-derived strain harbored the catalytic subunit *cdtB* critical for toxin activity. All strains carried six conserved capsule biosynthesis/transport genes (*kpsF, kpsD, kpsE, kpsM, kpsC*, and *Cj1419c*) and six LOS biosynthesis genes (*Cj1135, waaV, waaF, htrB, gmhA*, and *gmhB*). *C. jejuni* infection triggers GBS through molecular mimicry, where LOS induces antibody cross-reactivity with peripheral nerve gangliosides, leading to autoimmune-mediated nerve damage. Moreover, four GBS-associated genes were identified: 44 strains harbored *wlaN*, 23 carried *cstIII*, 39 possessed *neuA1*, and 27 exhibited *neuB1*, with 22 strains demonstrating co-occurrence of all four genes. *virD4* gene is one of the T4SS system genes, only B71 carried it. Distribution patterns of the remaining strain-specific virulence genes are presented in Figure 2.

### MLST and phylogenetic analysis

Forty-seven strains of *C. jejuni* alleles were uploaded to PubMLST to obtain sequence types (STs) and clonal complexes (CCs) as Supplementary Table 4 showed. Forty-seven strains were classified into 30 STs, including 3 novel STs (ST-14053, ST-13889, and ST-13892), 39 of them belonged to 10 CCs, and the remaining 8 strains were not assigned to any CC. CC-443 was the most frequent strain, accounting for 21.3% (10/47), followed by CC-574, accounting for 19.1% (9/47).

**FIGURE 3:**
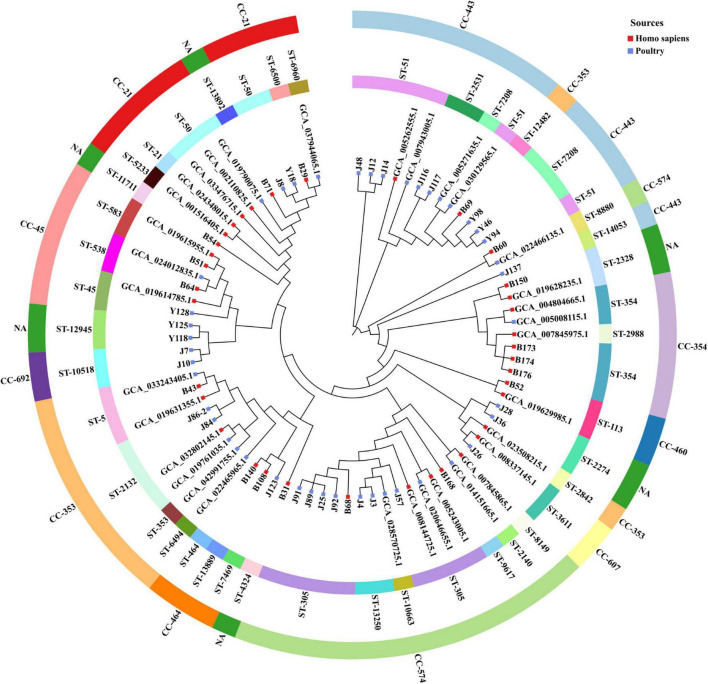
Phylogenetic tree of 80 *C. jejuni* isolates, including 47 isolates from this study and 33 reference isolates from the NCBI database. Outer ring blocks represent the CCs, inner ring blocks represent the STs. Blue blocks indicate poultry-derived strains, red blocks indicate *Homo sapiens*-derived strains.

To investigate the epidemiological origins and transmission of *C. jejuni*, we performed a core genomic phylogenetic analysis of 47 isolates in our study and 33 reference genomes from NCBI databases (Supplementary Table 5). As shown in Figure 3, the majority of poultry-derived strains did not form phylogenetic clusters with reference strains, while eight strains (B29, B43, B51, B52, B60, B64, B150, and J26) in our study showed close genetic relatedness with reference strains. Yet, only B60 and B29 demonstrated high genetic homology with Zhejiang (GCA_022466135.1) and Beijing (GCA_037944065.1) poultry-derived strains, suggesting the potential local transmission between human and poultry. Three CC-354, ST-354 strains B173, B174, and B176 exhibited high genetic homology with the local outbreak strain GCA.007845975.1 (CC-354, ST-2988).

## Discussion

In recent years, due to the increasing AMR of *C. jejuni* and the frequent occurrence of food poisoning incidents caused by it, more and more regions have included it in their surveillance systems. According to literature reports, the infection rate of *C. jejuni* in diarrhea cases in Shanghai (Shu et al., 2021), Beijing (Hai-bo et al., 2020), and other places (Peichao et al., 2023) in China is about 9%–14%. The detection rate of *C. jejuni* in poultry meat was about 26%–58%. The previous monitoring results of this study (cannot be cited) showed that the *C. jejuni* infection rate of diarrhea cases was 11.8%, and the *C. jejuni* detection rate of poultry meat was 46.5%, which was close to the reported results, and the infection risk should not be ignored. To our knowledge, this represents the first comprehensive genomic surveillance study in Hangzhou, employing high-density sampling during 2022–2023 to achieve improved detection rates of *C. jejuni* isolates obtained from both poultry sources and patients with diarrheal diseases. Therefore, understanding the genomic characteristics of *C. jejuni* in this area can provide a reference for disease early warning and prevention.

In this study, AMR of 47 strains of *C. jejuni* isolated from diarrhea patients and poultry samples in Tonglu District of Hangzhou were analyzed. Human-source *C. jejuni* exhibited six resistance profiles, whereas poultry-origin strains showed nearly double the diversity (*n* = 11), indicating broader antimicrobial selection pressure in poultry production systems. Quinolones-resistance and tetracycline-resistance are *C. jejuni* dominant resistance phenotypes (Moser et al., 2020; Meistere et al., 2019), which were also confirmed in our study. The results showed that the resistance rates of 47 strains of *C. jejuni* to quinolone antibiotics NAL and CIP were 93.6% and 91.5%, respectively, followed by TET 78.7%, showing high resistance. With the abuse of antibiotics and the gene mutation, *C. jejuni* has developed MDR. In our study, compared with human isolates, the AMR rate of *C. jejuni* isolated from animals was higher and multiple, drug resistance is even more serious. There were 9 different multi-drug resistance combinations, 22 strains of poultry-derived *C. jejuni* were resistant to 3 or more antibiotics, and among them, 8 strains belonging to CC-574 were resistant to at least 4 antibiotics. We found 18 strains from patients’ sources or raw poultry meats showed NAL + CIP + TET (18/47), followed by NAL + CIP + FLO + TET, 23.4% (11/47). It is suggested that NAL + CIP + TET is the most popular resistance pattern, consistent with other reports (Zhang P. et al., 2020; Wang et al., 2022), and this pattern occurred in the diversity of CCs, including CC-353 (3/18), CC-354 (3/18), CC-21 (2/18), CC-45 (2/18), etc. Meanwhile, combined with previous reports (Ju et al., 2018; Zhou et al., 2016), FLO resistance will be a new antimicrobial trend, FLO resistance rate reached 42.6% (20/47). Four strains belonging to CC-574 had NAL + CIP + FLO + TET AMR phenotype.

Further genetic analysis, a total of 6 classes of 25 drug-resistant genes were screened, which had a certain correlation with the drug-resistant phenotype but were not completely consistent. According to literature reports (Bush et al., 2020; Lin et al., 2005), quinolones resistance is mainly related to specific point mutations in the Quinolone Resistance Determination Region (QRDR) of DNA cyclase. The mutation of threonine 86 in *gyrA* to isoleucine is the most common, and a few studies (Ghielmetti et al., 2023) have found that the mutation of threonine 86 to alanine can lead to nalidixic acid resistance. Notably, we found that J26 exhibited the *gyrA* (*T86V*) mutation, also showed a high level of quinolone resistance (NAL: MIC ≥ 64, CIP: MIC = 32). To our knowledge, this mutation had been reported in majority *Campylobacter lari* with quinolone resistance (Jurinović et al., 2023), while a ciprofloxacin-resistant *C. jejuni* from German harbored *gyrA* (*T86V*) (Zeller-Péronnet et al., 2023). This rare mutation in *C. jejuni* suggested horizontal gene transfer or homologous recombination events may occurred.

Tetracycline resistance represents a significant threat to public health (Grossman, 2016), 78.7% (37/47) *C. jejuni* in this study were resistant to it. Tetracycline antibiotics reversibly bind to the 30S ribosomal subunit, inhibiting bacterial protein synthesis by blocking aminoacyl tRNA attachment to the A site, while Tet(O), an elongation factor-like protein, promotes drug release from the ribosome, conferring resistance (Grossman, 2016). This study confirmed that strains with tetracycline resistance genes have tetracycline resistance phenotypes, among which *tet (O)* (80.9%, 38/47) is the most common, along with mosaic genes *tet(O/M/O)* and *tet(O/32/O)*, suggesting potential horizontal gene transfer events.

Florfenicol, a synthetic monofluorinated derivative of thiamphenicol, is a new broad-spectrum antibiotic of chloramphenicol for veterinary use that was successfully developed in the late 1980s (Tang et al., 2017). More and more bacteria (*Escherichia coli*, *Staphylococcus sciuri*, *Klebsiella pneumoniae*) displayed florfenicol-resistance due to irrational usage of it (Li P. et al., 2020). Recent genomic surveillance data (up to 2023) from global public database indicate persistently low florfenicol resistance rates among *C. jejuni* isolates worldwide (Jia et al., 2025). However, a 2016 study in Beijing reported a rising trend in florfenicol resistance among *C. jejuni* isolates, with resistance rates increasing from 12% (1997–1999) to 62% (2009–2010) (Zhou et al., 2016). In our study, 7 human-origin and 13 poultry-origin *C. jejuni* strains were found to be resistant to florfenicol, overall rate was 42.6% (20/47) which exceeding global levels, yet no resistance gene was detected. The emergence of florfenicol resistance in *C. jejuni* may pose a serious global public health concern. As reported in Taiwan (Liao et al., 2022), 11.2% of *C. jejuni* were resistant to florfenicol, but only 1.1% of *C. jejuni* carried the resistance gene *fex, cfr (C)*. *C. jejuni* detected in Zhejiang in 2018 and 2019 also had phenotypes with high resistance to florfenicol, while no florfenicol resistance genes [*cfr(C)* and *RE-cmeABC*] were detected (Chen et al., 2020). Moreover, with the increasing severity of florfenicol resistance, phenotypic resistance appears more obvious than genotypic resistance, potentially due to other mechanisms or multi-drug efflux pumps (e.g., CmeABC efflux pumps).

In this study, the observed resistance rate to aminoglycosides (17%, comprising one human-derived and six poultry-derived *C. jejuni* isolates; see Supplementary Table 2) was notably lower compared to other antimicrobial classes. As shown in Zhou et al.’s (2016) report, the aminoglycoside resistance rate has been rising significantly. Notably, all poultry-derived *C. jejuni* isolates in our study exhibited STR resistance. Genetic analysis revealed that STR-resistant isolates (J91, J89, J25, B68, and Y118) consistently carried the *APH(3′)-IIIa* and *ant(6)-Ia* resistance genes. Consistent with previous reports (Bai et al., 2024; Deblais et al., 2023; Quino et al., 2021; Bravo et al., 2021), *APH(3′)-IIIa* was identified as the predominant aminoglycoside-resistant gene in human-derived and poultry-derived *C. jejuni*, whereas *ant(6)-Ia* was more prevalent in *C. coil*. Our findings suggested potential horizontal transfer of *ant(6)-Ia* resistance gene from *C. coli* to *C. jejuni.* However, J92 harbored both *APH(3′)-IIIa* and *ant(6)-Ia* genes remained aminoglycoside-sensitive, while J116 demonstrated phenotypic resistance yet lacking detectable aminoglycoside resistance genes. Similarly, J7 carrying the *aadE-Cc* gene exhibited STR resistance whereas J10 remained susceptible. Y125 maintained aminoglycoside sensitivity despite possessing the *ant(6)-Ia* gene. These discrepancies suggest potential undetected resistance mechanisms or regulatory variations influencing gene expression in specific isolates.

Since the Resistance Monitoring System for Enteric Bacteria (NARMS) does not include *Campylobacter* β-lactams, the kit purchased does not test for β-lactams drugs. β-Lactamase, a group of bacterial enzymes that hydrolyze the β-lactam ring, resulting resistance to this important antibiotic. *Campylobacter* species are considered intrinsically resistant to β-lactam antibiotics, making these drugs non-recommended for clinical treatment and reducing the need for routine surveillance of this resistance phenotype (Marotta et al., 2019). Most *C. jejuni* isolates exhibited a high prevalence of β-lactam resistance primarily mediated by β-lactamase production, with strong resistance against ampicillin and amoxicillin. However, susceptibility to other β-lactam antibiotics (e.g., imipenem, meropenem, ertapenem, and cefotaxime) remains possible (Casagrande Proietti et al., 2020; Griggs et al., 2009). Through genetic data analysis, nearly half of *C. jejuni* harbored class D β-lactamase (OXA) genes (Tooke et al., 2019) mediating resistance to β-lactam antibiotics. *blaOXA-61*, *blaOXA-193*, *blaOXA-450*, *blaOXA-451*, *blaOXA-452*, *blaOXA-453*, and *blaOXA-489* were found in 22 strains of *C. jejuni* mainly belonging to CC-574, CC-21, and NA complex. β-Lactamase expressed by the *blaOXA-61* gene is one of the most common and important cause of *Campylobacter* resistance to β-lactams. In the Indonesian study (Yanestria et al., 2023), all strains were resistant to aztreonam and 73.9% were resistant to ampicillin, all of them carried the *blaOXA-61* gene. In a study of *C. jejuni* in Ecuador (Montero et al., 2024), 47% of *C. jejuni* carried *blaOXA-193*, 15% carried *blaOXA-460*, along with other β-lactamase family genes. In other countries (Rokney et al., 2020; Deblais et al., 2023), *C. jejuni* WGS results can also find a variety of β-lactamases, and *blaOXA-193* proportion was also relatively prevalent. To some extent, our findings suggest the extensive genetic adaptation of *C. jejuni* to β-lactam pressure.

The CmeABC efflux pump, belonging to the resistance-nodulation-cell division (RND) family, is the predominant efflux system in *C. jejuni*. It comprises three structural components, a periplasmic fusion protein (CmeA), an inner membrane efflux transporter (CmeB), and an outer membrane protein (CmeC) (Iovine, 2013). The expression of CmeABC is transcriptionally repressed by CmeR, a regulatory protein encoded by the gene located upstream of *cmeA*. As a critical MDR mechanism, CmeABC actively extrudes diverse antimicrobials including fluoroquinolones, macrolides, and tetracycline (Tang et al., 2017; Lin et al., 2002). Mutations in *cmeR* or the intergenic region (IR) located between *cmeR* and *cmeA* can upregulate cmeABC expression to increase the level of resistance to antimicrobial (Lin et al., 2005). Seventy-four *C. jejuni* isolates from diarrhea patients with IR mutation displayed higher resistance rates to fluoroquinolones, tetracyclines, florfenicol, chloramphenicol, and gentamicin (Yang et al., 2017). The CmeA plays a critical role in the efflux pump system. In Mu et al.’s (2013) study, PNA-mediated inhibition of either *cmeA* or *cmeB* significantly reduced bacterial resistance to CIP and ERY, while simultaneous inhibition of both genes further enhanced susceptibility to these antibiotics. As for CmeB, structural impairment of CmeB has been shown to diminish resistance to multiple antibiotics (Lin et al., 2002). However, the RE-CmeB variant, which shares only 81% sequence identity with wild-type CmeB demonstrates markedly elevated MDR phenotypes (Yao et al., 2016). In our study, *cmeB* was undetectable in 24 isolates, among *cmeB*-negative isolates, 20 exhibited resistance to florfenicol, 19 to tetracycline, 21 to nalidixic acid, and 20 to ciprofloxacin. In contrast, *cmeB*-positive isolates displayed resistance limited to quinolones and tetracyclines. This phenotypic divergence suggests that the *cmeB* variants undetected in database may exhibit significant sequence divergence, similar to *RE-cmeB*, where structural alterations potentially enhance MDR phenotypes, and we suggested florfenicol-resistance was strongly associated with RE-CmeABC efflux pump.

*Campylobacter* causes disease mainly through adhesion, colonization, invasion, movement and toxin secretion, which is the result of co-expression of various virulence factors. In addition, it also includes some specific function genes such as LOS related genes and type IV secretion system genes (Bravo et al., 2021; Quino et al., 2021). A total of 106 virulence genes in 7 classes were detected in this study, adherence genes (*cadF, pebA, jlpA*, and *porA*) and invasion genes (*ciaB* and *ciaC*) carrying rates were 100%, suggesting these *C. jejuni* had early potential pathogenicity. The *cadF* gene is responsible for adhesion in host cells, detected in reports from Brazil (Moreira Lima et al., 2022; Melo et al., 2019) and India (Iqbal et al., 2024), *cadF* gene detection rate was 100%. *Campylobacter* Invasion Antigens (*Cia*) secretion by Type III Secretion System (T3SS) triggers *C. jejuni* uptake by intestinal epithelial cells, but the detailed mechanism is not fully understood (Tikhomirova et al., 2024). With the same reports in other countries, *ciaB* and *ciaC* had a relatively high detection rate, 96% and 100% in Ecuador (Montero et al., 2024), 95.5% *ciaB* in Brazil (Melo et al., 2019).

Motion-related genes, including those involved in flagella synthesis, chemotaxis, and motility accessory factors, were extensive, with 43 genes detected in all isolates and the remaining 11 genes showing carrying rates ranging from 34.0% to 97.9%. *flaA* and *flaB* encoding the filament of flagella are important genes for motility. However, we found not all *C. jejuni* carried *flaA* (83%, 39/47) and *flaB* (72.3%, 34/47), consistent with the report to *C. jejuni* of poultry origin in Brazil between 2015 and 2016 (Melo et al., 2019). Chemotaxis is the significant function of *C. jejuni* which can facilitate it move to favorable conditions or escape from detrimental ones (Tikhomirova et al., 2024). We found chemotaxis factor genes (*cheW*, *cheV*, and *cheY*) were detected in all *C. jejuni*, but *cheA* was not harbored.

Cytolethal distending toxin is considered a major cause of pro-inflammatory response in campylobacteriosis (Tikhomirova et al., 2024), encoding with *cdtA, cdtB*, and *cdtC*. The carrying rates of *cdtA* and *cdtC* were 100% and 97.9%, respectively. *cdtB*, as the main virulence gene and active unit, cooperated with binding units *cdtA* and *cdtC* to cause disease. In this study, only one human strain carried *cdtB* gene. Genes related to capsular synthesis and LOS were also detected. *kpsM* is related to *C. jejuni* capsular synthesis, helping it adhesion to and invasion of human intestinal epithelial cells (Tikhomirova et al., 2024), *kpsM* carrying rate was 100% in our study. Studies have reported that LOS genes are associated with GBS (Tikhomirova et al., 2024), in this study, 14 kinds of LOS related genes were detected, and the carrying rates of *Cj1135, waaV, waaF, htrB, gmhA*, and *gmhB* were 100%. In particular, the carrying rates of *wlaN, cstIII, neuA1*, and *neuB1* virulence genes related to GBS (Fernández-Palacios et al., 2024; Hull et al., 2021) were 93.6%, 48.9%, 83.0%, and 57.4%, respectively. These results suggest that human-derived and poultry-derived *C. jejuni* isolates have strong pathogenic potential in this area. Notably, strain B71 uniquely harbored *virD4* on the plasmid *pVir*, consistent with previous reports (Quino et al., 2021), suggesting that *virD4* may not be a critical virulence factor, but is an accessory virulence gene influenced by horizontal gene transfer.

The epidemiology of *C. jejuni* in this study exhibited notable genetic diversity, characterized by dispersed STs across multiple CCs. CC-443 was the predominant clonal complex (10/47, 21.3%), followed by CC-574, accounting for (9/47, 19.1%), highlighting their potential role in local transmission. Notably, CC-443 has recently been identified as a dominant strain among the cold-tolerant strains isolated from retail raw chicken (Hur et al., 2022). Considering the dietary habits and consumption patterns prevalent in our region and across the nation, the purchase of raw poultry stored in cold chains is a common practice. It poses a critical food safety risk due to the potential pathogen transmission between retail poultry and humans. Intriguingly, CC-574, which accounted for only 0.9% in the PubMLST database (May 2025), emerged prevalently in our region. Compared with reference ST-305 strains, local ST-305 strains formed a distinct phylogenetic subcluster (Figure 3), suggesting the localized microevolution under regional selective pressures. Similarly, CC-460 and CC-692, representing 0.6% and 0.3% in PubMLST, respectively, were also detected locally. In contrast, CC-21 the second most prevalent clonal complex globally (16.3% in PubMLST, May 2025) was represented by only three strains in this study, showing distinct regional epidemiological patterns compared to global distributions. In this study, three CC-354 strains isolated from diarrhea patients clustered on the same phylogenetic branch as a *C. jejuni* strain isolated from human stool (NCBI accession: GCA_007845975.1) from the 2018 foodborne outbreak in Hangzhou, implying the presence of undetected pathogen reservoirs in the local environment. Notably, this clade also included poultry-associated strain from United States (NCBI accession: GCA_005008115.1), aligning with the hypothesis of poultry-to-human transmission. Additionally, B69 (CC-353, ST-12482) formed a subclade with CC-443 strains (Y94, Y46, and Y98). B71 (CC-NA, ST-13892) clustered closely with CC-21 strains. Similarly, B69 (CC-353, ST-12482), GCA_022466135.1 (CC-574, ST-8880), and CC-443 were clustered together, indicating that there are genetic relationships in the core genome, the method of numbering strains through seven housekeeping genes still has limitations.

## Conclusion

In conclusion, *C. jejuni* isolated from human stool and poultry sources between 2022 and 2023 in Hangzhou showed high MDR and diverse genetic types with many virulence factors. Resistance to quinolones and tetracycline was prevalent, up to 91.5% and 78.7%, respectively, driven by mutations in *gyrA*(*T86I*) and novel *gyrA*(*T86V*) and *tet(O)*. However, these genotypes did not exactly match the antimicrobial phenotypes, as 20 strains showed florfenicol resistance without any related resistance genes being detected. AMR results suggested quinolones and tetracycline resistance remain the dominant threats, while florfenicol resistance may become a new one. CC-464 and CC-21 strains carried the most virulence genes, correlating with clinical severity, yet represented a minority in local isolates. Virulence factor genes were prevalent in *C. jejuni* in Hangzhou, with CC-443 and CC-574 predominating in poultry, implicating raw poultry products as key transmission hosts. Thus, these findings underscore the public health threat of multidrug-resistant with hypervirulent strains. Enhanced surveillance is necessary to monitor the poultry-to-human transmission pathways and emerging resistance in high-risk clones.

## Data Availability

The original contributions presented in this study are included in this article/Supplementary material, further inquiries can be directed to the corresponding author.

## Footnotes

1
https://pubmlst.org/bigsdb?db=pubmlst_campylobacter_seqdef

2
https://www.chiplot.online/

3
https://card.mcmaster.ca/

4
http://genepi.food.dtu.dk/resfinder

5
http://www.mgc.ac.cn/VFs/main.htm
